# Unravelling complex choices: multi-stakeholder perceptions on dialysis withdrawal and end-of-life care in kidney disease

**DOI:** 10.1186/s12882-023-03434-5

**Published:** 2024-01-03

**Authors:** Chandrika Ramakrishnan, Nathan Widjaja, Chetna Malhotra, Eric Finkelstein, Behram Ali Khan, Semra Ozdemir, STEP Study Group, Chetna Malhotra, Eric Finkelstein, Behram Ali Khan, Semra Ozdemir, Jason Chon Jun Choo, Boon Wee Teo, Althea Chung Pheng Yee, Hua Yan, Vincent Wei Xiong See

**Affiliations:** 1 Duke-NUS Medical School, Lien Centre for Palliative Care, Programme in Health Services and Systems Research, Singapore, Singapore; 2 Duke NUS Medical School, Signature Programme in Health Services and Research, Singapore, Singapore; 3 Nanyang Technological University, Singapore, Singapore; 4 Department of Population Health Sciences, Duke University, Durham, NC USA; 5 Division of Nephrology, National University Health System, Singapore, Singapore; 6 National Kidney Foundation, Singapore, Singapore; 7 Duke Clinical Research Institute, Duke University, Durham, NC USA

**Keywords:** Decision-making, Dialysis withdrawal, Palliative care, End-stage kidney disease, The Ottawa Decision Support Framework

## Abstract

**Background:**

For patients on dialysis with poor quality of life and prognosis, dialysis withdrawal and subsequent transition to palliative care is recommended. This study aims to understand multi-stakeholder perspectives regarding dialysis withdrawal and identify their information needs and support for decision-making regarding withdrawing from dialysis and end-of-life care.

**Methods:**

Participants were recruited through purposive sampling from eight dialysis centers and two public hospitals in Singapore. Semi-structured in-depth interviews were conducted with 10 patients on dialysis, 8 family caregivers, and 16 renal healthcare providers. They were held in-person at dialysis clinics with patients and caregivers, and virtually via video-conferencing with healthcare providers. Interviews were audio-recorded, transcribed, and thematically analyzed. The Ottawa Decision Support Framework’s decisional-needs manual was used as a guide for data collection and analysis, with two independent team members coding the data.

**Results:**

Four themes reflecting perceptions and support for decision-making were identified: a) poor knowledge and fatalistic perceptions; b) inadequate resources and support for decision-making; c) complexity of decision-making, unclear timing, and unpreparedness; and d) internal emotions of decisional conflict and regret. Participants displayed limited awareness of dialysis withdrawal and palliative care, often perceiving dialysis withdrawal as medical abandonment. Patient preferences regarding decision-making ranged from autonomous control to physician or family-delegated choices. Cultural factors contributed to hesitancy and reluctance to discuss end-of-life matters, resulting in a lack of conversations between patients and providers, as well as between patients and their caregivers.

**Conclusions:**

Decision-making for dialysis withdrawal is complicated, exacerbated by a lack of awareness and conversations on end-of-life care among patients, caregivers, and providers. These findings emphasize the need for a culturally-sensitive tool that informs and prepares patients and their caregivers to navigate decisions about dialysis withdrawal and the transition to palliative care. Such a tool could bridge information gaps and stimulate meaningful conversations, fostering informed and culturally aligned decisions during this critical juncture of care.

**Supplementary Information:**

The online version contains supplementary material available at 10.1186/s12882-023-03434-5.

## Background

The global incidence and mortality rate of end-stage kidney disease (ESKD) are rising, particularly among older adults [1]. In Singapore, ESKD-related deaths have increased by 44.3% between 2011 and 2020 [2], a trend expected to continue due to an ageing population, high diabetes prevalence [3], and low kidney transplant rates [4].

While dialysis is an effective first-line treatment for ESKD, evidence suggests that it can pose significant challenges and stress for patients as their quality of life and prognosis deteriorate and symptom burden increases after years of undergoing dialysis [5]. Consequently, international guidelines recommend consideration of dialysis withdrawal, often referred to as discontinuation of dialysis, and the transition to end-of-life (kidney supportive care or palliative care) care [6, 7]. This care approach prioritizes symptom management and focuses on patient-oriented quality of life [8–10]. However, the decision to withdraw from long-term dialysis is complex and can be overwhelming due to the ethical and emotional nature of the decision [11, 12].

While there is a growing recognition of dialysis withdrawal for patients with poor quality of life and poor prognosis, considerable disparities exist in dialysis withdrawal rates across different regions globally [10, 13]. These variations can be attributed in part to divergent attitudes towards end-of-life care, which are influenced by cultural and religious differences among societies [14]. Most existing studies and guidelines have predominantly emanated from and focused on Western countries [11], potentially rendering their recommendations unsuitable to populations with diverse cultural backgrounds [14]. For example, the medical decision-making processes in Western societies are centered around individual autonomy while in Asian cultures, medical decisions are often highly influenced or led by families [14, 15].

Furthermore, existing studies, with the notable exception of Russ et al.’s study [12], have primarily focused on perspectives of individual stakeholders in isolation (i.e. patients [16–18], caregivers [19, 20], or healthcare providers (HCPs) [21–23]), or limited dyadic perspectives (i.e. patient-HCPs [24–26], or patient-family [27]) while neglecting the comprehensive triadic viewpoints. In addition, previous research has primarily focused on advance care planning, reasons for stopping dialysis, or end-of-life experiences regarding dialysis patients. However, there is a notable gap in the literature investigating the decision-making process and informational needs for making decisions regarding dialysis withdrawal from the perspectives of all stakeholders involved in decision-making.

In Singapore, although legislation addresses the termination of non-beneficial treatments [28] and advances have been made in the quality of palliative care planning and provision [29], cultural factors such as the taboo surrounding death [30, 31], strong familial influence [32, 33] and paternalistic medical decision-making [34] continue to hinder patient autonomy and informed decision-making regarding dialysis withdrawal and transition to end-of-life care. This qualitative paper aims to explore the triadic perspectives of patients on dialysis, their family caregivers, and renal HCPs in order to identify their information needs and evaluate the support required for decision-making regarding dialysis withdrawal and the adoption of end-of-life care.

## Methods

The qualitative study followed the Consolidated Criteria for Reporting Qualitative Studies (COREQ) [35] guidelines for reporting (Supplement 1).

### Setting and participants

Participants were recruited from the National Kidney Foundation (NKF), a not-for-profit organization operating dialysis centers across Singapore, and two major public hospitals: Singapore General Hospital and National University Hospital. The study included patients on hemodialysis (HD), family caregivers of HD patients, caregivers of deceased HD patients, and renal HCPs.

Eligible patients were identified from the medical records of the HD patients (referred to as patients henceforth) at eight dialysis centres. The inclusion criteria included being 21 years or older (the age of majority in Singapore), currently undergoing dialysis, being cognitively intact (assessed by an abbreviated mental test, by a physician or from medical records), agreeable to audio recording, and meeting one of the following: a) prognosis of fewer than 12 months as determined by a treating physician at the dialysis clinic using the surprise question: *Would I be surprised if this patient died in the next 12 months?*, b) tolerating dialysis poorly (physically or mentally) as identified by a treating physician at the dialysis clinic, or c) expressed a desire to discontinue dialysis.

Informal caregivers of eligible patients were eligible for the study if they were aged 21 years or older and provided care, ensured the provision of care, or made decisions regarding patient care without expecting financial compensation. Spouses and adult offspring caregivers were sampled to obtain diverse perspectives. Caregivers of deceased patients within 6 months of death were also included to enrich the data with their experiences regarding dialysis withdrawal and end-of-life care.

Eligible patients and caregivers were contacted in person or over the telephone by two medical social workers. Out of 44 participants approached (34 patients and 10 caregivers), 26 (24 patients and 2 caregivers) declined participation due to lack of interest, sensitivity to the topic, or lack of time.

HCPs were recruited from three participating institutions based on the following criteria: being a nephrologist, palliative care physician, renal nurse, renal counsellor, medical social worker, or clinical psychologist currently providing care to dialysis patients. HCPs were nominated by department heads and invitation letters were sent via email. Among 18 HCPs approached, two did not respond while the rest agreed to participate.

### Data collection

A semi-structured interview guide was developed based on the Ottawa Decision Support Framework (ODSF) decisional needs manual [36]. The ODSF guides researchers to assess and address decisional support needed for difficult decisions. The interview guides (Supplement 2) tailored for each stakeholder group, were reviewed by the study steering committee comprising HCPs (2 nephrologists, 2 medical social workers, 1 renal nurse, 1 renal counsellor), and 2 patient- and 1 caregiver-representatives.

In-depth interviews were conducted between February and October 2022. All interviewers were trained in conducting qualitative interviews and they had no previous relationship with the study participants. Patient and caregiver interviews were conducted in-person in English by the first author or in Chinese by two team members fluent in the language, in a quiet private room at their preferred place (dialysis centre or their homes). Patients and caregivers were interviewed separately to enable conversations to be open and candid. HCP interviews were conducted remotely over a video conferencing platform in English by the first and corresponding authors. The interviews lasted 20–60 min and were audio-taped, transcribed verbatim and (Chinese interviews) translated, and repeat interviews were not conducted. Field notes were summarized following the interview. Recruitment continued in conjunction with analysis, and data collection ended when no new information or ideas were generated.

### Data analysis

Thematic analysis was conducted using the framework proposed by Braun and Clarke in nVivo11 [37]. Our deductive analysis and coding were based on the ODSF decisional needs manual [38]. The initial set of codes was derived a priori based on the ODSF operational and conceptual definitions. Two team members independently reviewed the transcripts and assigned sections of text to the pre-defined codes, and texts were mapped to the codes. Code categories were discussed during team meetings, and any discrepancies were resolved by a third team member. Themes and sub-themes were developed deductively from the ODSF framework. Periodic meetings were held until consensus on salient themes and sub-themes was reached among team members. Exemplar quotes were extracted to illustrate these themes. Data collection continued alongside data analysis until no new themes emerged. Participant review of transcripts was not included, as we had a priori codes that could potentially alter the interpretation of data.

### Ethical considerations

Written informed consent was obtained from all participants. The study was approved by the National University of Singapore, Institutional Review Board (Ref no NUS-IRB-2021–749).

## Results

A total of 34 individuals (10 patients, 8 caregivers, and 16 HCPs) participated in the study ranging from 31–80 years with 59% females. The participant characteristics are detailed in Table 1.

**Table 1:** Participant characteristics (*N* = 34)

	**Healthcare providers** ***n* = 16**	**Patients** ***n* = 10**	**Caregivers** ***n* = 8**
**Age range,** years, n (%)			
31–40	12 (75)	0	1 (12.5)
41–50	2 (12.5)	1 (10)	1 (12.5)
51–60	2 (12.5)	1 (10)	2 (25)
61–70	0	4 (40)	2 (25)
71–80	0	4 (40)	2 (25)
**Sex**, Female, n (%)	12 (75)	2 (20)	6 (75)
**Ethnicity,** n (%)			
Chinese		5 (50)	5 (62.5)
Malay		2 (20)	2 (25)
Indian		2 (20)	0
Others		1 (10)	1 (12.5)
**Education,** n (%)			
Primary		3 (30)	3 (37.5)
Secondary		5 (50)	1 (12.5)
Junior college		2 (20)	3 (37.5)
University		0 (0)	1 (12.5)
**Stakeholder type,** n (%)	Nephrologist 5 (31)	On hemodialysis: 10 (100)	Caregivers of current patients: 5 (62.5)
	Palliative care physician: 1 (6)		Caregivers of deceased patients: 3 (37.5)
	Renal nurse: 3 (19)		
	Medical social worker: 4 (25)		
	Renal counsellor: 2 (13)		
	Clinical psychologist: 1 (6)		
**Occupation,** n (%)			
Home maker		1 (10)	0
Not working		5 (50)	0
Working part-time		1 (10)	2 (25)
Working full-time		0 (0)	4 (50)
Retired		3 (30)	2 (25)

**Fig. 1:**
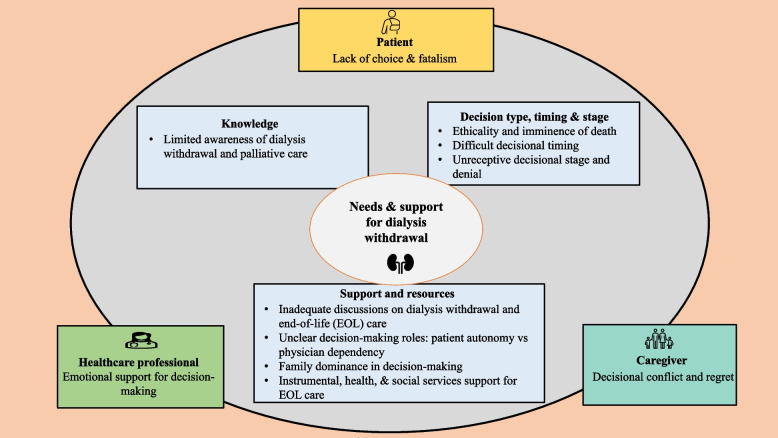
Decision-making needs and support for dialysis withdrawal and end-of-life care

Overall, four main themes were identified. Table 2 presents the themes, subthemes, and minimally-edited verbatim extracts. Figure 1 presents a visual representation of these themes at the stakeholder level**.**

**Table 2:** Themes and subthemes with illustrative quotes on information needs and support for decision-making for dialysis withdrawal

Themes	Sub themes	Illustrative quotes
**Poor knowledge and fatalistic perceptions**	Perceptions of having no choice but to continue dialysis	*Due to the dialysis, I feel very down. Honestly, very sad. I mean the family is in this problem…. it’s very difficult (to cope). I don’t know, because sometimes I am so bored of my life. Sometimes I give up. I know (it is) my life. At this stage (of my dialysis), I'm not happy. I have to continue to do (dialysis) every week 3 times- Monday, Wednesday that’s all I can remember that’s why I forgot the day I forgot the time. Very sad*. Patient/PT04/F *I’ve been sitting down all the time (for dialysis), it's very inconvenient. I try to finish and go back early. That's my concern. Then sometimes, I get so fed up and tell them (nurses) “Oh,,I don’t want to do (dialysis) already, I die, I don’t mind.” You know very, very discouraging. So just happened to get angry so I just say that, but of course I know I have to do.* Patient/PT09/M *I have no choice (but to continue dialysis). If I don't do it (dialysis), what can- what am I going to do? Can I do anything? So, I have to do it. I have to tolerate it.* Patient/PT09/M *No choice, once you get this kind of illness (kidney failure) you must continue.* Caregiver (spouse of PT07)/CG04/F *Unfortunately, those who started many years back, many of them felt that it’s like a death sentence on dialysis and they didn't know there was this option (to stop dialysis) so when I do financial assistance for patients where they are on maintenance dialysis, it’s interesting, they will always tell me I didn't know there's this option to stop.* Clinical psychologist/HCP11/F
	Limited awareness regarding dialysis withdrawal and palliative care	*My thinking is this (dialysis) is a lifelong procedure to me. As long as I live I have to go for dialysis. I do not try to stop (dialysis)…. I haven’t heard about it stopping dialysis really.* Patient/PT03/M *To me, this kind of thinking (about stopping dialysis) is very childish [laughs] So I won’t- won’t say these kinds of things. Because I know, if you don’t continue, it’s the end for you already.* Patient/PT05/M *Every week if you need to dialyse three days you have to come three days. You can’t say like you want to come two days, one day, cannot lah. Cannot be unreasonable like that.* Patient/PT06/M *These things (Palliative care) for dialysis, I didn’t know they had these. I knew they had it for cancer, because my brother-in-law received this palliative (care)…But for dialysis, nobody talked to us about it.* Caregiver (Daughter) of Deceased/CG08/F *I never come across (end-of-life care) ah. It is the first time I am hearing all this.* Caregiver/CG01/M *And if they (patients) choose supportive care basically, they are not aware that they will be taken care by us (medical team) as well. They would think, and a lot of them feel that they are left on their own.* Nurse/HCP04/F *Because the major shortcoming in the past years and even currently is that patients do not even know that they have the right to stop their own dialysis. The areas of deficit really are in the knowledge, the awareness and the empowerment.* Nephrologist/HCP06/M
**Inadequate information and support for decision-making**	Inadequate discussions regarding dialysis withdrawal and end-of-life care	*I’ve never thought about this (stopping dialysis). He (doctor) doesn’t say so much.* Patient/PT06/M *My doctor in XX hospital, the renal doctor is very busy one. She also has got no time for all these (discussions on EOL care) things.* Patient/PT09/M *Unless the doctor gives us proper knowledge, what will happen if you stop this (dialysis) and what benefit the patient under this very good. But so far, I never heard (of dialysis withdrawal).* Caregiver (spouse of PT04) /CG01/M *Not a single doctor told us that her condition was like that, they only asked us if we had ever considered stopping dialysis. […] There was no continuation to say, have you guys considered these details about her illness, what would happen to her in future, how much she would suffer. […] If it was just a bit earlier, we had known that her condition was like that, (if) we had understood that (dialysis) really brought her so much harm, we would have- maybe we would have considered stopping her dialysis earlier.* Caregiver (Daughter) of Deceased/CG08/F *I think it's the culture and it is a culture that we don't start to bring this up to patients. And this is something that they (patients) will not express you know that ‘I want to die peacefully’,…because to them (patients) you know to bring up the word death is something not good and not something they will accept. So that's why they also don't bring up (these conversations)*. Nurse/HCP10/F *Singapore is a very Asian society. We actually don’t talk much about terminal care and withdrawal of dialysis.* Nephrologist/HCP12/M *Yeah, this is something that I see, or know there's a lack of awareness amongst the patient, because it's not so, at least this is not so openly talk about, you know, if unless you know they are close to the end and all that…It could be the discomfort of thinking that you know the death is so, so close. You know it's it. There's this certain fear. Yeah, they haven't really accepted that, you know, this is coming.* Medial social worker/HCP07/F *Some training of the physicians and facilitators who run these discussions is going to be very helpful because not everybody can manage this kind of discussion. You know I just kind of learned how to do it, I kind of figured my way out. But, I've been around a fair number of years. So, so that helps a lot I think, but not everybody can manage this*. Nephrologist/HCP06/M
	Unclear decision-making roles: Patient autonomy vs physician dependency	*I really considered my wife’s decision in terms of my dialysis. Because they have to decide, cause one thing, one thing is they have to pay. Yeah. In my decisions in life, I consider their- their decision much more than my decision.* Patient/PT03/M *Instead of you know nobody even my husband dare not make decision on behalf of me. I myself, I make the decision. You are not allowed to make decision on behalf of myself. Because I don’t want to. The person who would suffer to answer all this is myself. So I told my son whatever the doctor tell you just say I couldn’t make decision, my mother would make.* Patient/PT04/F *First thing is to get information from the doctors. Before that you need to talk to the nurse first after that doctor. The doctor will have the final decision for the stopping of dialysis.* Patient/PT01/M *If the patient has mental capacity, then, of course, still patient’s decision is most important. I would rather like mentioned earlier I would organize a family conference for them. For patient who express to everyone why did he or she wants to stop dialysis, what is in his mind…. I will say patients’ values, really, very important, because ultimately all medical decision is a joint decision making. We do listen to what patients are thinking.* Nephrologist/HCP05/F *There's the last group that actually will just listen to essentially what a medical professional say and they will just follow through. You know, actually, is not easy to, especially on physicians, to tell the patient, that eh you know it's time to stop because I think that is always a certain degree of responsibility on the physician because (it) is a therapy and you want the patient to live as long as possible.* Nephrologist/HCP02/M
	Caregiver dominance in decision-making	*I keep it (my feelings about dialysis withdrawal) from them, but they know, they know. They would know… They know that my MSW know. My wife knows, but, uh, I don't- we don't talk about it, but they know* Patient (spouse of CG03)/PT03/M *Unless she is very very stressful, she is all to give up, I say cannot. We have to advice her life has to go on. If you don’t go for dialysis how are you going to survive. This is not like any other sickness.* Caregiver/CG01/M *And sometimes I don't want to talk about his sickness if possible, because I don't want him to become hopeless and depressed about the sickness.* Caregiver (spouse of PT03)/CG03/F *From my experience, they (family caregivers) will, they will brush it off. As in, because they have heard it from the patient themselves before. So the patient will also tell their family members, aiyah I am very tired, I want to stop. I don't I don't want to go dialysis anymore. But then the patient has to keep going for dialysis. So the family hears, hears this many times already. So when MSW tells the family this, the family will just say ‘aiyah he’s like that lah, he just say, say, only you know he just say, talk about it. Right, So, so that's kind of a brushing off or dismissing the feeling. Yeah, that's my experience.* Medical social worker/HCP08/M *We tell them (caregivers) what the each of their, you know their loved one feels and what they say. We do tell them that we're going to share what you said to us to the patient as well. And we don't get a lot of resistance, a lot of times and they're willing because they just can't talk to the patient and they want us to do the job. But we tell them that you know we can only do our best to relay messages, but we hope that you can also listen to what your loved one has to say.* Nurse/HCP04/F *You know, Singapore society is very different than (Western societies), we are, we are very driven by family. […] A lot of times, even though they decide for the good of the patient, that decision may not be what the patient actually wanted. […]But sometimes you just automatically see that family member bulldoze away over the patient.* Nephrologist/HCP02/M
	Emotional support for decision-making	*Personally, my own opinion is, I think it is good to always lay the cards all on the table and tell the person, you know, what would happen if let's say dialysis is stopped, you know, what she can expect, you know, or what would be the procedure, what would be done and all that. And if she's very clear, you remove the fear (of the unknown) from her, then maybe she'll be more receptive because at least she knows, what is going to happen.* Caregiver (Sister) of Deceased/CG07/F *I think it is more of emotional needs that they really need [to be addressed]. Not so much of social but lot of emotional and moral support that they need from the family members, and I think also from us- the dialysis centre staff you know to continue, to just have conversation with them so that they (patients) know that we do care although they are at their end of life. And we are doing whatever we can to maintain those (who stopped dialysis) so that they can pass on peacefully.* Nurse/HCP10/F *After patient pass away, we also follow up on their (caregiver) grief and if they require support, we make referrals to the family service center counsellors for the follow up with the family members if they consent*. Medical Social worker/HCP14/F
	Instrumental, health and social services for supporting end-of-life care	*Maybe for myself, I need to know about the support for me about my care and if I come to that stage, how to do the things with help from somebody else. Do your every (day) things, you have to pee or pass motion or what- whatsoever. Cause you are already in the bed. The medical, medical support.* Patient (spouse of CG03)/PT03/M *We were also made aware, because we told the doctors there that we didn't want my sister to die in my house because I wouldn't know how to handle it, you know, it would be a very terrifying thing for me.* Caregiver (sister) of deceased/CG07/F *I think most important is to let them (patients) know that they are not abandoned even if they choose to stop dialysis.* Medical social worker/HCP03/F *We find out that it (dialysis withdrawal) is a well-considered decision (for a patient), then we will provide information on hospice, palliative options like hospice, or home hospice, and we may even ask the doctor, our XXX doctor whether this patient is suitable for fewer dialysis fewer times per week…. Then, I will do a referral to the hospital to MSW where they have their palliative team, their palliative doctor and nurses can set up a support system for the patient and family at their home when the patient has totally stopped dialysis*. Medical social worker/HCP08/M
Complexity of decision-making, unclear timing, and unpreparedness	Dialysis withdrawal is a difficult decision	*You see everyone on dialysis, everyone comes out alive and well, it’s very good! I think it over, if I stop, what can I do? I will die what to do? There’s nothing can be helped already what. You don’t do dialysis, then what are you going to do? Wait to die?* Patient/PT09/M *I cannot say that (stop dialysis) because it is too risky. If you stop, how is she (patient) going to survive? Its better unless she has gone through all this for years then suddenly you stop, then it’s like try to kill somebody.* Caregiver (Spouse)/CG01/M *It’s just like suicide like that. He’ll definitely die if he doesn’t do dialysis*. Caregiver (spouse)/CG02/F *You know it’s a very difficult choice because we are talking about life and death now, literally. And they are talking about stopping them, and that means their life is ended, their relationship with a family’s gone you know and, and the way they know of life is terminated. So, it is a very sensitive and tough topic for the patients, especially those that have not really thought about it.* Nephrologist/HCP02/M *Because we, of course, then would bring in very naturally the concern on the part of both caregivers, patients and physicians of euthanasia, which is currently not legal in Singapore. You know, patients and families often misperceive stopping a medical therapy a life sustaining medical therapy as euthanasia killing the patient actively. This is a very difficult area to clarify.* HCP06_Nephrologist
	Unclear decisional timing	*When she (the patient) just started dialysis ah, patients who just started dialysis would- maybe they wouldn’t have these kinds of ideas (of stopping dialysis) to think that they would pass on so quickly. Maybe say after dialysing for a period of time, or three- three or four years later, you have to look at the patient’s condition (before deciding when to discuss dialysis withdrawal).* Caregiver (Daughter) of Deceased/CG08/F *But I do hope that you know, it will start at the point where any patients or every patient that come in or before even starting on dialysis or when they know that their kidneys not functioning. To know that there is always support all the way until the end, and supportive care should be discussed. Much, much earlier. So we actually start from the beginning all the way to the end rather than you know when you are in dire straits and then you come to us and there is only so much I can hold your hand for.* Nurse/HCP04/F *If they come in the outpatient clinic setting, they will have quite a lot to say about their suffering, which can be multi-domain from physical to psychosocial. So, there is usually what they focus on, and then we try and address all these things because all these things could be confounding their decision to stop dialysis and sometimes after we address some of these they actually continue (dialysis). So, the request to stop dialysis, is kind of a cry for help, rather than request to really withdraw (from) the treatment.* Clinical Psychologist/HCP13/F*But I would say it's always a stepwise procedure in a sense that it is difficult to actually right at the start to let the patient know that dialysis withdrawal is on cards. I guess it's important to first make the patient realize that we are struggling with dialysis. And I think a lot of times is a topic that you have to actually repeatedly visit, where you may not actually be able to get through to the patient of the family at the first instance and it requires in fact I will say several discussions before all parties may be able to come to terms with the decision.* Nephrologist/HCP15/F *I think it (withdrawal) should be considered maybe in the earlier stage when a patient is still able to make his or her own decision and able to express clearly, able to express the values. And because the moment we start, there will be a stop. So, I think this kind of discussion (for dialysis withdrawal) maybe should be done earlier before the patient went into some form of critical illness and medically unfit already.* Nephrologist/HCP05/F *I have only counted very few patients who have electively decided to cease dialysis when they are not critically ill. One of these, was a very insightful spouse and family, where the patient had severe dementia and increasingly could not interact as an individual. So, the family and spouse felt that the patient had lost his dignity. So, they electively decided to cease dialysis, even though the patient was not critically ill.* Nephrologist/HCP06/M
	Unreceptive decisional stage and denial	*Nothing, nothing. I mean, once come here- life is normal. that’s all. You already used to it (dialysis) already, so there's no other way to change.* Patient/PT08/M *On top of her dialysis session, she also was- I could see that she was very reluctant to give up her life which every one of us also will be reluctant, you see. It was a very I would say that it was a very sad moment, but all of us accepted the fact that we eventually had to help, to let her see that death may actually be a better choice, no point dragging on because (of) the dialysis.* Caregiver (Sister) of deceased/CG07/F *Most patients, I will say maybe 60 to 70%, they do have considered, and they know about this option (to stop dialysis). But majority still feels is important to live on, so they don't think (deliberate) about the withdrawal so much*. Renal counsellor/HCP16/F *It could be the discomfort of thinking that you know the death is so close. You know it is. There's this certain fear, they (patients) haven't really accepted that you know, this (end of life) is coming, and they may not be so open….‘I don't think I am at that stage yet’.So you know sometimes they (patients) just brush us off.* Medical social worker/HCP07/F
**Internal emotions of decisional conflict and regret**	Decisional conflict	*He told us that we really had to think it through, the feelings of the patient, because for us to help her (mother) decide these kinds of things, I thought that it was cruel. I asked myself every time, if I make this decision, is it like I’m murdering her (mother), it was really like you would feel like you were killing her. From another angle, the doctor’s perspective, actually it’s not like that. Actually, it’s because you don’t want to let her suffer, you want her to be relieved from this suffering, you want to think about it on her behalf, she would suffer a lot if she (continued) dialysis every day. ‘Have you thought about her (mother’s) suffering?’ She (doctor) said that her blood pressure was rising and falling every day, and the burden on her heart. I thought about all these, that’s why I considered giving up. If not, we didn’t- because we wouldn’t know exactly whether our decision was right or wrong*. Caregiver (Daughter) of deceased/CG08/F *In the situation if their (family’s) loved one is not able to advocate for themselves about wanting to with regards to terminating dialysis, then they will feel and be afraid to make that decision. So, usually it goes back to the doctor to make that decision.* Medical social worker/HCP14/F *And they (caregivers) struggle with a lot ‘am I doing the right thing and am I making the right decision or am I pushing too hard. Should I just respect, am I giving up without a fight for my loved ones, you know, and letting him (loved one) make a decision for not doing dialysis on too easily’. I think these are some of the internal struggles that they (caregivers) have to deal with on top of the resource, finance, money issues or care issues that they have to deal with day in and day out*. Clinical psychologist/HCP11/F *Depending on how the medical team and how they approach the topic, the families may feel that they have to be the ones to make the decision. That's when they (family) feel guilty because you know the moment they say yes to stopping (dialysis), then they have a direct role because they know that the prognosis is so short. So, they (family) may sense that and they feel like they have a direct role in hastening the death or causing the death of a loved one.* Palliative care physician/HCP13/F
	Regret for a delayed decision to withdraw dialysis	*When we held on to her for so long, just two days ago, I asked my dad, “Did we make a mistake, should we have stopped her dialysis earlier? Did we do something very, very selfish by letting her (continue dialysis).- Why didn’t we think about her properly, about how exactly her condition, when her condition was at its most severe, why didn’t we think about giving up?* Caregiver (Daughter) of deceased/CG08/F *But deep down we know that she believed that she would be going to a better place. And we kept telling her that, yes, if you are going to believe in that, then we support your belief, you are going to a better place. Any place is definitely (better) than your suffering now, isn't it? When you are already suffering like that, right? Any place that has got no pain is a blessing……I worried so much….I will choose quality. If let’s say, you know, it comes to a stage where, I’m in such pain and I have to worry so much, then I'd rather let go… My sister prolong and prolong until this year, then she passed away three months ago* Caregiver (Sister) of deceased/CG07/F

### Theme 1: Poor knowledge and fatalistic perceptions

#### Perceptions of having no choice but to continue dialysis

Many patients and caregivers viewed dialysis as a lifeline, making it difficult to consider dialysis withdrawal under any circumstances. Patients often held fatalistic beliefs that they had “no choice” but to continue with dialysis “until they died”. Only a minority of patients mentioned that they would consider dialysis withdrawal due to treatment fatigue. HCPs noted that patients and caregivers often did not completely comprehend the possibility that continuing dialysis may be medically futile or untenable in the future.

#### Limited awareness regarding dialysis withdrawal and palliative care

Most patients and caregivers were unaware that dialysis withdrawal was a patient’s medical right and a viable option. They were unsure of available palliative care options following dialysis withdrawal. For instance, one caregiver believed that palliative care was exclusively for cancer. Consequently, some patients and caregivers feared being “left on their own”, or believed that they would suffer more if they stopped dialysis. As such, some patients associated dialysis withdrawal with “childish” thinking or “unreasonable” non-compliance. HCPs reported that most patients lacked “knowledge, awareness, and empowerment” to make decisions regarding dialysis withdrawal.

### Theme 2: Inadequate support and resources for decision-making

#### Inadequate discussions regarding dialysis withdrawal and end-of-life care

Patients and caregivers often expressed a lack of conversations with their physicians about disease progression and end-of-life care. HCPs were perceived as “very busy” and “have no time” for in-depth discussions. Notably, almost all patients and caregivers reported that their physicians had not broached the possibility of dialysis withdrawal. Most patients reported not having advance care planning discussions, with some indicating a preference for ‘nature to take its course’.

HCPs reported several barriers to initiating these discussions, including a cultural taboo regarding death. To illustrate, a physician noted, “Singapore is a very Asian society. We do not talk much about terminal care and withdrawal of dialysis.” Furthermore, HCPs noted that discussions about end-of-life care preferences “is not something that they [patients] would volunteer” due to a "certain fear of death". They also anticipated resistance from patients and caregivers, as the concept of death was viewed as “not something they [patients and families] will accept”, rendering it as a rationale for why they “do not bring it up”. In addition, HCPs reported limited time for engagement with patients and a lack of skills in end-of-life communication.

#### Unclear decision-making roles: Patient autonomy vs physician dependency

Patients exhibited diverse preferences for decision-making roles regarding dialysis withdrawal. Some patients leaned towards shared decision-making, valuing discussions with their families and physicians. A subset of patients emphasized autonomy, viewing the decision as deeply personal and asserting that no one else should be “allowed to make a decision on behalf of myself”. Conversely, other patients preferred to delegate decision-making to their families, noting that “their [families] decision is much more important than my decision”. Alternatively, they entrusted their physicians with the responsibility, putting faith in their medical expertise and believing that physicians would act in their best interests. However, physicians instead preferred to provide objective medical information. They acknowledged the pivotal role of “patient’s values”, and emphasized that “patient’s decision is (the) most important” and “ultimately all medical decisions [should be] joint decision making”.

#### Caregiver dominance in decision-making

HCPs noted the significant influence caregivers have on patients, a dynamic that occasionally undermined patient autonomy. Some caregivers pressured patients to continue dialysis, noting that “we had to advise her [patient]; life had to go on”, even if this conflicts with patients' own desires. While caregivers have good intentions, HCPs noted that they often lacked understanding of patients’ fatigue and suffering on dialysis. This was articulated by an HCP’s observation that “a lot of times, even though they [families] decide for the good of the patient, that decision may not be what the patient actually wanted*”*.

Furthermore, some caregivers intentionally avoided discussing medical matters with patients to prevent them from feeling “hopeless and depressed” about their condition. Consequently, some patients felt more comfortable discussing dialysis withdrawal with HCPs rather than their caregivers. To navigate these communication barriers, HCPs sometimes assume the role of mediators between patients and caregivers. In particular, they felt compelled to advise caregivers that “we can only do our best to relay messages, but we hope that you can also listen to what your loved one has to say”.

### Emotional support for decision-making

To support decision-making regarding dialysis withdrawal, HCPs emphasized the need to reassure patients and caregivers that, medical teams would “not abandon if they choose to stop dialysis” and continue to support patients in “passing on peacefully”. These include providing emotional assistance throughout patients’ EOL care, and preparing families for their loved ones’ imminent death, including bereavement support to cope with grief. A caregiver voiced that receiving such reassurance early on helped her loved one become “more receptive” towards dialysis withdrawal.

### Instrumental, health, and social services for supporting end-of-life care

Patients desired information and assurances regarding symptom management and “about the support for [their] care” after dialysis withdrawal. HCPs stressed the importance of providing information about community resources, financial assistance schemes, and comprehensive care plans that meet patients’ and caregivers’ needs. HCPs also emphasized preparing caregivers to help them handle the practical and emotional demands of EOL care at home. A caregiver of a deceased patient recounted that having her loved one die at home would have been a “very terrifying” experience. She was ultimately grateful that her loved one passed away peacefully in a hospice.

### Theme 3: complexity of decision-making, unclear timing, and unpreparedness

#### Dialysis withdrawal is a difficult decision

Another key theme was the ethicality and difficulty of deciding on dialysis withdrawal. Patients expressed that dialysis prolonged their life and the thought of dialysis withdrawal disturbed them due to the imminence of death. Caregivers often viewed dialysis withdrawal as “giving up” and one caregiver even likened it to "suicide". Many HCPs also acknowledged that dialysis withdrawal can be perceived as "choosing to die", "suicide", or "euthanasia", and added that discussing dialysis withdrawal is challenging due to these perceptions.

#### Unclear decisional timing 

Although HCPs agreed that decision-making regarding dialysis withdrawal should be a "step-wise procedure", involving multiple discussions, they seemed to disagree about the timing of initiating these discussions. While some HCPs thought these discussions should start earlier around dialysis initiation, others recommended that the "optimal time is when medical problems or recurring issues arise". HCPs narrated cases of patients having suicidal thoughts, such as "wanting to die", and "to stop dialysis and walk away from home". However, HCPs also emphasized that these thoughts may arise in the "fit of the moment" or as a way of "venting frustrations about the dialysis process". They viewed these thoughts as cues to further investigate whether dialysis withdrawal should be considered for the patient. HCPs expressed that palliative care teams should be included to "ease the patient into the discussions" and "explore psychosocial aspects" to ensure that dialysis withdrawal is a "well-considered decision".

#### Unreceptive decisional stage and denial

Patients generally showed resistance to discussing dialysis withdrawal. They reflected that they had not considered dialysis withdrawal and would only consider it as a last resort. Patients stated that "life was normal on dialysis", and there were "no other ways to change a life". Some HCPs also observed that patients often become comfortable with the dialysis routine over time and are reluctant to stop it even when it is no longer beneficial. HCPs highlighted that patients and caregivers are often not in a mindset to consider dialysis withdrawal, perceiving it as a far-off decision. One bereaved caregiver mentioned that her loved one was in denial and reluctant to consider dialysis withdrawal even after her physicians recommended it.

### Theme 4: internal emotions of decisional conflict and regret

#### Decisional conflict

Caregivers of deceased patients expressed being "unsure" or "afraid" when deciding whether to withdraw patients from dialysis, questioning the rightness or wrongness of their decisions. Caregivers whose loved ones ultimately underwent dialysis withdrawal reported that while it was a difficult and "cruel" choice, stopping dialysis turned out to be a "practical" choice that "relieved suffering". Physicians described the "guilt" that caregivers expressed when faced with a dialysis withdrawal decision, as they felt responsible for "hastening the death of their loved one".

#### Regret for a delayed decision to withdraw dialysis

Caregivers of deceased patients expressed feeling "selfish" for not fully considering their loved one’s perspective and regretting not making the dialysis withdrawal decision earlier. This internal conflict was exemplified by a caregiver’s reflection: “Did we make a mistake, should we have stopped her dialysis earlier”. Such introspections arose from a realisation that an earlier withdrawal might have reduced their loved one’s suffering. All caregivers of the deceased shared a dual emotional response following their loved one’s passing, marked by a feeling of sorrow in the face of a loss and a sense of relief that their loved one’s suffering had finally come to an end.

## Discussion

This study examined the triadic perspectives of patients on dialysis, their caregivers, and renal HCPs, with a specific focus on decision-making concerning dialysis withdrawal and the subsequent transition to end-of-life care in Singapore. The findings revealed that patients and caregivers had a limited understanding of dialysis withdrawal and palliative care. Notably, dialysis withdrawal was associated with feelings of abandonment, lack of support, and increased suffering. These findings are consistent with the previous studies showing that patients are often unaware of dialysis withdrawal [39] and available end-of-life care options [18], or perceive dialysis withdrawal as a form of medical abandonment [5, 40].

The complexity of decision-making regarding dialysis withdrawal was influenced by existential, ethical, and familial factors, hindering open discussions and informed decision-making. In parallel to extant findings [5, 11], patients and caregivers struggled to consider dialysis withdrawal due to the imminence of death following withdrawal. Instead of viewing it as a means to relieve suffering, dialysis withdrawal was often dismissed as “giving up” or even akin to a form of suicide. While some studies showed that patients may express interest in dialysis withdrawal due to declining quality of life [12, 18, 41], most patients in our study had become accustomed to their dialysis routines even though some expressed treatment fatigue and suicidal thoughts. Consequently, the idea of contemplating end-of-life care remained a distant consideration, denying the necessity to discuss it until continuing dialysis would become medically untenable.

This denial was reinforced by the lack of end-of-life conversations between patients and their clinicians and/or caregivers. Although some patients preferred a paternalistic approach to their care, HCPs hesitated to have these conversations due to cultural taboos regarding death, anticipated resistance from patients and caregivers, insufficient time, and lack of skills in end-of-life communication. This finding resonates with other local studies with renal HCPs showing low frequencies of discussions regarding advance care planning [42] and inadequate palliative care training [43]. HCPs also raised concerns about caregivers not being able to fully comprehend patients’ treatment burden, and sometimes over-riding patient preferences to prolong their lives. Furthermore, some caregivers avoided discussing medical issues with patients to prevent negative emotions. Consequently, these cultural and familial barriers, along with the lack of end-of-life discussions from HCPs, impeded informed decision-making.

Interviews with bereaved caregivers further revealed the complex nature of the decision. These interviews illuminated a range of emotions, including feelings of guilt stemming from not knowing how to proceed, regret for delaying dialysis withdrawal, and eventual relief following a peaceful death.

Overall, the research findings suggest that discussions and decision-making surrounding dialysis withdrawal and end-of-life care are primarily shaped by familial dynamics (e.g., dominant family role in decision-making), and cultural factors (e.g., taboo nature of discussing end-of-life issues), rather than individual factors (e.g., socio-economic status, age). In addition, institutional factors, such as lack of adequate time and training in communication skills for handling, emerged as significant factors contributing to the study outcomes.

Our findings unveil a complex web of emotional barriers among all stakeholders, hindering meaningful discussions and adequate support for end-of-life care and dialysis withdrawal. Patients reported grappling with the fear of abandonment and death, caregivers described the daunting prospect of being unable to meet their loved ones’ end-of-life care needs, while clinicians expressed concerns about causing distress or hastening death. These fears fuel a collective avoidance of conversations surrounding end-of-life care and dialysis withdrawal, possibly exacerbated by patients resorting to denial as a coping mechanism. Recognizing the profound effect of emotional challenges, we suggest interventions such as emotional counseling and support groups facilitated by allied health professionals. These initiatives can be used to address and alleviate the fears and anxieties experienced by patients and caregivers, fostering a more informed and emotionally prepared decision-making process.

Our findings underscore the imperative for HCPs to adopt a strategic approach that centers on delivering clear, compassionate, and culturally-sensitive information about dialysis withdrawal, especially the potential benefits and drawbacks of continuing versus stopping dialysis. To counteract the prevalent misconception that dialysis withdrawal equates to medical abandonment, it is crucial for HCPs to offer clarification and inform about unwavering support that would be available throughout palliative and end-of-life care. Given the ethical and emotional complexities surrounding dialysis withdrawal and end-of-life decisions, it is essential for HCPs to receive training in undertaking serious illness conversations and guidelines that facilitate better identification of patients who could benefit from these discussions. Building skills in effective communication, cultural competence, and addressing existential concerns can equip providers to guide patients and caregivers through these challenging decisions. HCPs should also be provided with guidelines to assess when patients and their families are ready to have these conversations.

Future research can focus on identifying the specific types of training for HCPs that are most effective in facilitating meaningful and effective end-of-life conversations. In addition, identifying patient preparedness for conversations centred around advance care planning in the context of dialysis withdrawal and the transition to end-of-life care is another avenue for investigation. This understanding could potentially lead to more personalized and effective communication strategies tailored to each patient’s unique emotional state and circumstances. These tools and training materials can all be part of a decision aid that can be developed to facilitate congruous and shared decision-making regarding dialysis withdrawal.

The study findings should be interpreted within the context of the study limitations. Findings derived from qualitative research are by nature prone to a degree of potential subjectivity. Despite efforts to engage a wide sample of stakeholders, we did not include patients on peritoneal dialysis and patients dialyzed at hospitals, who may have different perspectives and decisional needs.

The study has notable strengths. First, there is limited empirical research on the perspectives of multiple stakeholders on this topic. By including patients on dialysis, caregivers, and renal HCPs, as well as purposively sampling bereaved caregivers, we obtained a comprehensive understanding of the decision-making needs for dialysis withdrawal and experiences of EOL care. Second, the qualitative approach allowed us to examine the lived experiences and complexities associated with dialysis withdrawal decision-making[44]. Third, using the ‘surprise question’ helped identify a selective population of patients with poor prognoses who may face a decision to withdraw from dialysis in the near future. Last, we ensured study rigor in the methodology and reporting adhering to the COREQ guidelines. Additionally, incorporating a sound framework to guide systematic data collection and analysis enabled a more precise identification of decisional needs.

## Conclusions

Withdrawing from dialysis was viewed as a difficult decision due to the imminence of death and reluctance towards discussing end-of-life care. The lack of information and limited conversations regarding dialysis withdrawal and end-of-life care, coupled with disagreements regarding decision-making roles contributed to the complexity. While educational materials on dialysis withdrawal have been developed and utilized in Western contexts, there currently exists no decision aids specifically tailored for dialysis withdrawal. Our study highlights the importance of developing a culturally-sensitive educational and decision aid to prepare patients and their caregivers for dialysis withdrawal and transition to end-of-life care. These tools can also empower HCPs to initiate challenging yet crucial end-of-life conversations. The ultimate goal is to foster shared and informed decision-making, aligned with patient values, and sensitive to cultural contexts.

### Supplementary Information

**Additional file 1: Table S1.** Consolidated criteria for reporting qualitative studies (COREQ) 32-item checklist.

** Additional file 2.** Interview guide.

## Data Availability

The study data are available on reasonable request from the corresponding author subject to approval from the institution, funder and ethics review board.

## Abbreviations

ESKD: End-stage kidney disease
DW: Dialysis withdrawal
EOL: End of life
HCP: Healthcare provider
HD: Hemodialysis
ODSF: Ottawa decision support framework
COREQ: Consolidated criteria for reporting qualitative studies
