# Supplementary material for: Unravelling complex choices: multi-stakeholder perceptions on dialysis withdrawal and end-of-life care in kidney disease
Source: BMC Nephrol. 2024 Jan 3;25:6. doi: 10.1186/s12882-023-03434-5 (PMC10765633; doi:10.1186/s12882-023-03434-5)
Supplement: Supplementary file 1 — Additional file 1: Table S1. Consolidated criteria for reporting qualitative studies (COREQ) 32-item checklist. [file 12882_2023_3434_MOESM1_ESM.docx]

**Supplement 1: Consolidated criteria for reporting qualitative studies (COREQ) 32-item checklist**

| **No** | **Item** | **Guide questions/description** | **Performed by** |
| --- | --- | --- | --- |
| **Domain 1: Research team and reflexivity** | | | |
| **Personal Characteristics** | | | |
| 1. | Interviewer/facilitator | Which author/s conducted the interview or focus group? | English interviews: Chandrika Ramakrishnan (CR-first author);  Semra Ozdemir (SO- senior author)  Mandarin interviews: Research team member Jia Jia Lee (JJL)-mentioned in the acknowledgment section;  Nathan Widjaja (NW-second author) |
| 2. | Credentials | What were the researcher's credentials? *E.g. PhD, MD* | CR-MBBS MPH  JJL- MPH  NW- student  SO- PhD |
| 3. | Occupation | What was their occupation at the time of the study? | CR - Research Fellow  (no clinical duties)  JJ- Research Associate  NW-Undergrad student  SO - Assistant Professor |
| 4. | Gender | Was the researcher male or female? | NW- Male  CR, JJL, SO - Female |
| 5. | Experience and training | What experience or training did the researcher have? | Training and experience in qualitative research methods, including conducting in-depth interviews, coding, and thematic analysis |
| **Relationship with participants** | | | |
| 6. | Relationship established | Was a relationship established prior to study commencement? | No relationship |
| 7. | Participant knowledge of the interviewer | What did the participants know about the researcher? e*.g. personal goals, reasons for doing the research* | The participants know the reason for the research- i.e. it was about the perceptions on decision-making for dialysis withdrawal and end-of-life care. |
| 8. | Interviewer characteristics | What characteristics were reported about the interviewer/facilitator? e.g. *Bias, assumptions, reasons and interests in the research topic* | Their experience with conducting qualitative interviews and that they had no relationship with the study participants.  Reasons for doing research: to understand perceptions on decision-making for dialysis withdrawal and end-of-life care |
| **Domain 2: study design** | | | |
| **Theoretical framework** | | | |
| 9. | Methodological orientation and Theory | What methodological orientation was stated to underpin the study? *e.g. grounded theory, discourse analysis, ethnography, phenomenology, content analysis* | Thematic analysis framework underpinned by the Ottawa Decisional Support Framework (ODSF) |
| **Participant selection** | | | |
| 10. | Sampling | How were participants selected? *e.g. purposive, convenience, consecutive, snowball* | Purposive sampling was used.  Eligible patients were identified by screening medical records by physicians at NKF dialysis centers using a checklist (inclusion criteria). The physicians who screened are not the usual treating physicians but are stationed at dialysis centers to attend to patients when needed. A list of eligible patients meeting at least one of the inclusion criteria was handed over to the social worker for recruitment. |
| 11. | Method of approach | How were participants approached? e*.g. face-to-face, telephone, mail, email* | Eligible patients on dialysis and their caregivers were approached face to face or by telephone by two social workers (study team); healthcare providers were approached over email |
| 12. | Sample size | How many participants were in the study? | 22 participants attended one-to-one interviews |
| 13. | Non-participation | How many people refused to participate or dropped out? Reasons? | 28 participants declined participation due to a lack of interest, sensitivity to the topic, or lack of time.  No one dropped out. |
| **Setting** | | | |
| 14. | Setting of data collection | Where was the data collected? e*.g. home, clinic, workplace* | Private room in the healthcare setting or virtual (over Zoom) |
| 15. | Presence of non-participants | Was anyone else present besides the participants and researchers? | No |
| 16. | Description of sample | What are the important characteristics of the sample? *e.g. demographic data, date* | Reported in Table 1 |
| **Data collection** | | | |
| 17. | Interview guide | Were questions, prompts, guides provided by the authors? Was it pilot tested? | The topic guide was created by the authors. Yes, pilot testing was done with study advisory board members (including patients on dialysis and caregivers of patients on dialysis) |
| 18. | Repeat interviews | Were repeat interviews carried out? If yes, how many? | No |
| 19. | Audio/visual recording | Did the research use audio or visual recording to collect the data? | Audio recordings |
| 20. | Field notes | Were field notes made during and/or after the interview or focus group? | Yes, after the interview |
| 21. | Duration | What was the duration of the interviews or focus group? | Interviews lasted 20 to 60 minutes |
| 22. | Data saturation | Was data saturation discussed? | Yes |
| 23. | Transcripts returned | Were transcripts returned to participants for comment and/or correction? | No |
| **Domain 3: analysis and findings** | | | |
| **Data analysis** | | | |
| 24. | Number of data coders | How many data coders coded the data? | 2 |
| 25. | Description of the coding tree | Did authors provide a description of the coding tree? | Yes, the codebook was created using the Ottawa Decisional Support Framework – decisional needs coding manual |
| 26. | Derivation of themes | Were themes identified in advance or derived from the data? | Derived from data |
| 27. | Software | What software, if applicable, was used to manage the data? | QSR NVivo 11 |
| 28. | Participant checking | Did participants provide feedback on the findings? | No |
| **Reporting** | | | |
| 29. | Quotations presented | Were participant quotations presented to illustrate the themes / findings? Was each quotation identified? e*.g. participant number* | Yes, the quotes are presented with the participant number |
| 30. | Data and findings consistent | Was there consistency between the data presented and the findings? | Yes |
| 31. | Clarity of major themes | Were major themes clearly presented in the findings? | Yes |
| 32. | Clarity of minor themes | Is there a description of diverse cases or discussion of minor themes? | Yes |
