# Supplementary material for: Unravelling complex choices: multi-stakeholder perceptions on dialysis withdrawal and end-of-life care in kidney disease
Source: BMC Nephrol. 2024 Jan 3;25:6. doi: 10.1186/s12882-023-03434-5 (PMC10765633; doi:10.1186/s12882-023-03434-5)
Supplement: Supplementary file 2 — Additional file 2. Interview guide. [file 12882_2023_3434_MOESM2_ESM.docx]

**Supplement 2: Interview guide**

**Patients on dialysis**

**Introductory questions**

Could you tell us a bit about yourself and your family?

Can you tell me a bit about your living situation?

Does anyone help you with eating, changing, toileting or bathing?

**Experience with dialysis**

How long have you been on dialysis?

What type of dialysis are you currently undergoing?

Can you describe to me a typical day when you get dialysis?

What problems do you face in doing dialysis?

Can you tell me about the cost of medical treatments?

How are you feeling these days?

**Stopping dialysis: awareness and information needs**

Have you ever thought of or feel like stopping dialysis? If yes, why

Have you discussed this with your doctor? Or with your family?

Has your kidney doctor ever mentioned/discussed stopping dialysis with you? If yes, Can you remember what they discussed with you?

Have you had any counselling with a medical social worker?

Did you look for (other) information about stopping dialysis?

Under what circumstances/situations will you/someone consider stopping dialysis?

Are you aware of options/what to do if you (someone) if dialysis is not helping you anymore?

Have you heard of palliative or supportive care or hospice care?

Has someone discussed Advance care planning with you?

What kind of information/support/resources do you think you will need in order to decide on stopping dialysis at some point?

If you or any dialysis patient were to receive counselling or information about stopping dialysis, what kind of information will be helpful?

Who would be the main decision maker when you are considering stopping dialysis?

What is most important to you (what do you value most) in the last stages of your life?

How do you see yourself spending your last weeks/months of your life?

**Caregivers**

**Introductory questions**

To start, could you tell us about your role as a caregiver?

What responsibilities do you have as caregiver?

**Experience with dialysis**

How long has your [spouse, mother, father, etc.] been on dialysis?

What type of dialysis your [spouse, mother, father, etc.] receives?

Can you describe to me a typical day when your [spouse, mother, father, etc.] gets dialysis?

Do you know if your [spouse, mother, father, etc.] are experiencing any side-effects from dialysis?

What are the changes you have had to make in your daily living recently due to your [spouse, mother, father, etc.]’s condition?

What support do caregivers like you need?

**Stopping dialysis: awareness and information needs**

Have you ever thought of your [spouse, mother, father, etc.] stopping dialysis? If yes, why?

Have you discussed this with your loved one? Or with his/her doctors?

Do you know or heard about anyone who has considered stopping dialysis?

Has your [spouse, mother, father, etc.] ever talked/discussed with you about stopping dialysis? If yes, why?

Has the kidney doctor ever mentioned/discussed stopping dialysis with you or your [spouse, mother, father, etc.]?

If yes, can you remember what they discussed with you or your [spouse, mother, father, etc.]?

Has you or your [spouse, mother, father, etc.] had any counselling with a medical social worker about stopping dialysis?

Did you look for (other) information about stopping dialysis?

Under what circumstances do you think you or your [spouse, mother, father, etc.] or someone will consider stopping dialysis?

Are you aware of options/what to do if your [spouse, mother, father, etc.] or someone stops dialysis?

What kind of information/support/resources do you think he will need in order to decide on stopping dialysis?

Where do you think a patient would prefer to get information about stopping dialysis?

If you were to receive counselling with a decision aid on stopping dialysis, what type of information will be helpful?

Who would make the main decision maker when your [spouse, mother, father, etc.] is considering stopping dialysis?

What is most important to your [spouse, mother, father, etc.] in the last stages of his/her life?

**Healthcare providers**

What is your role in helping patients on dialysis?

For how long have you been doing this?

When a patient is not doing well on dialysis (dialysis is not helping the patient any more) and has a poor prognosis, what are the common concerns you hear from them regarding dialysis?

In your practice, how often do you come across situations when you would recommend patients (and their caregivers) to consider stopping dialysis? (For clinicians only)

Have you come across a situation when a patient on dialysis expresses the desire to stop dialysis? How about their caregiver?

Is this common? What are the main reasons for patients wanting to stop dialysis?

Can you tell me a bit more about the decision-making dynamics between patients and their caregivers?

How often do you see disagreement between patients and their caregivers about stopping dialysis?

What are the care options available for patients after dialysis withdrawal?

Do you discuss about palliative/supportive care with dialysis patients and/or caregivers? (for physicians and nurses)

Based on your experience, what do you think are the emotional and social needs of patients on dialysis who should consider stopping dialysis?

What information do you provide to patients on dialysis who need to make a decision on continuing or stopping dialysis?

Do patients express their personal values? E.g. dying at home, quality of life, etc.
